# Case Report: Parvovirus B19 infection complicated by hemophagocytic lymphohistiocytosis in a heart-lung transplant patient

**DOI:** 10.3389/fimmu.2023.1099468

**Published:** 2023-02-07

**Authors:** Xuewu Zhang, Jingxia Wang, Xiaohan Huang, Yue Zhu, Yijing Zhu, Lingling Tang, Hongliu Cai, Xueling Fang, Lingtong Huang

**Affiliations:** ^1^ Department of Critical Care Units, The First Affiliated Hospital, Zhejiang University School of Medicine, Hangzhou, China; ^2^ Department of Hematology, The First Affiliated Hospital, Zhejiang University School of Medicine, Hangzhou, Zhejiang, China; ^3^ Zhejiang Provincial Key Laboratory of Hematopoietic Malignancy, Zhejiang University, Hangzhou, Zhejiang, China; ^4^ Department of Infectious Diseases, The First Affiliated Hospital, Zhejiang University School of Medicine, Hangzhou, Zhejiang, China; ^5^ Department of Nephrology, The First Affiliated Hospital, Zhejiang University School of Medicine, Hangzhou, China; ^6^ Department of Infectious Diseases, Shulan (Hangzhou) Hospital, Zhejiang Shuren University of Shulan International Medical College, Hangzhou, China; ^7^ Key Laboratory of Clinical Evaluation Technology for Medical Devices of Zhejiang Province, Hangzhou, China

**Keywords:** parvovirus B19, transplantation, HLH, hemophagocyticsyndrome, hemophagocytic lymphohistiocytosis

## Abstract

Immunosuppressed patients can contract parvovirus B19, and some may experience hemophagocytic lymphohistiocytosis (HLH). Herein, we describe the first report of hemophagocytic lymphohistiocytosis in a heart-lung transplant patient with concomitant parvovirus B19 infection. The patient was treated with intravenous immune globulin (IVIG) and the features of HLH were remission. This instance emphasizes the significance of parvovirus B19 monitoring in transplant patients with anemia; if HLH complicates the situation, IVIG may be an adequate remedy. Finally, a summary of the development in diagnosing and managing parvovirus B19 infection complicated by HLH is provided.

## Highlights

- Parvovirus B19 infection complicated by HLH is uncommon in transplant patients
- IVIG is an effective treatment for parvovirus B19 infection complicated by HLH

## Introduction

Parvovirus B19 is an ancient and conserved virus that circulated 100 million years ago or earlier (1). It is associated with pure red cell aplasia (PRCA) (2–4), viral myocarditis (5–8), erythema infectiosum (9), and other clinical manifestations. At the same time, evidence of the presence of parvovirus B19 has also been found in bone marrow transplant recipients (10) and diseases such as systemic lupus erythematosus (11, 12), miscarriage (13), systemic sclerosis (14), hereditary hemolytic anemias (15). Infectious erythema is one of the most common clinical manifestations of parvovirus B19 infection, which often occurs in children (4). Parvovirus B19 infection induced PRCA may present severe anemia and reticulocytopenia (4). Viral reactivation can occur in proerythrocytes and myocardial cells, and could be the cause of multi-organ damage (4–8). The pathogenic effects of parvovirus appear to be immune-mediated (5–8, 11, 12, 14, 15). Besides, the expansion of viral inclusion bodies in proerythroblasts mediating erythroid maturation arrest has also been observed in PRCA patients suggesting the direct pathogenic effect of the virus (2, 16). Intravenous immune globulin (IVIG) may be effective for PRCA (3, 4), intrauterine anemia (17), mantle cell lymphoma (18) due to the presence of IgG-neutralizing antibodies against parvovirus B19. However, the efficacy of IVIG is still unclear for viral myocarditis (19) and chronic fatigue syndrome (20) associated with parvovirus B19.

HLH is a group of rare but life-threatening disorders characterized by hyperinflammatory responses and dysregulated immune cells. There are many causes of HLH, including inborn errors of immunity, inborn errors of metabolism, and many kinds of tumors, including lymphoma (21). A variety of viral infections can trigger HLH (22), including human herpesvirus and human immunodeficiency virus (23). Less commonly, parvovirus B19 is associated with the life-threatening HLH; hence, early identification of triggers and treatment of the primary disease is key to a good prognosis.

There are few case reports of parvovirus B19 infection complicated by HLH in transplant patients (24–26). Herein, we describe a case of HLH in a heart-lung transplant patient due to parvovirus B19 infection. Through IVIG treatment alone, the maturity of the erythroid was recovered, and the features of HLH were in remission. Finally, we summarize the reported cases of parvovirus B19 infection complicated by HLH.

## Case presentation

**Figure 1:**
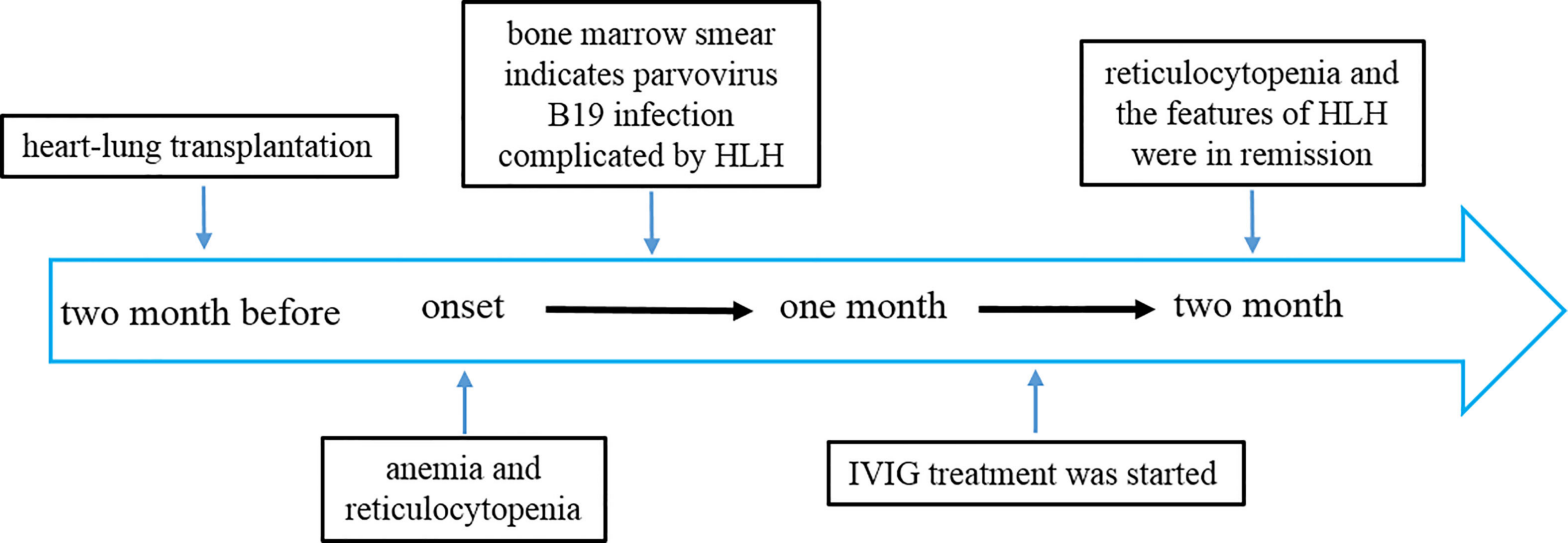
Timeline depicting the disease course of the patient. The timeline illustrates the different events in the course of the patient’s treatment and disease progression.

A 59-year-old female suffering from heart and lung failure due to long-term pulmonary hypertension underwent cardiorespiratory combined transplantation and was given tacrolimus and methylprednisolone for anti-rejection after transplantation. She had no other medical history, and no hereditary illnesses ran in her family. The patient had no bleeding from the wound and no acute rejection after the operation. She received two months of rehabilitation. Two months later, her condition changed, and she experienced repeated reductions in hemoglobin (60 g/L, reference range 130-170 g/L) and reticulocytes (0.001×10^12^/L, 0.1%) (**Figure 1**).

Anemia was not improved after symptomatic support treatment. The monitoring of biochemical showed alanine aminotransferase, glutamic oxaloacetic aminotransferase, bilirubin, creatinine, and myocardial enzyme were within the normal range which indicated that there was no organ dysfunction of liver and kidney. During this period, although the patient had repeated fever, pathogenic tests of blood culture, sputum culture, urine culture and pleural effusion culture were all negative. Her C-reactive protein was 1.4 mg/L (reference range 0-8 ng/mL) and procalcitonin was 0.08 ng/mL (reference range 0-0.5 ng/mL) which suggested common pathogens were unlikely to be the cause of anemia.

**Figure 2:**
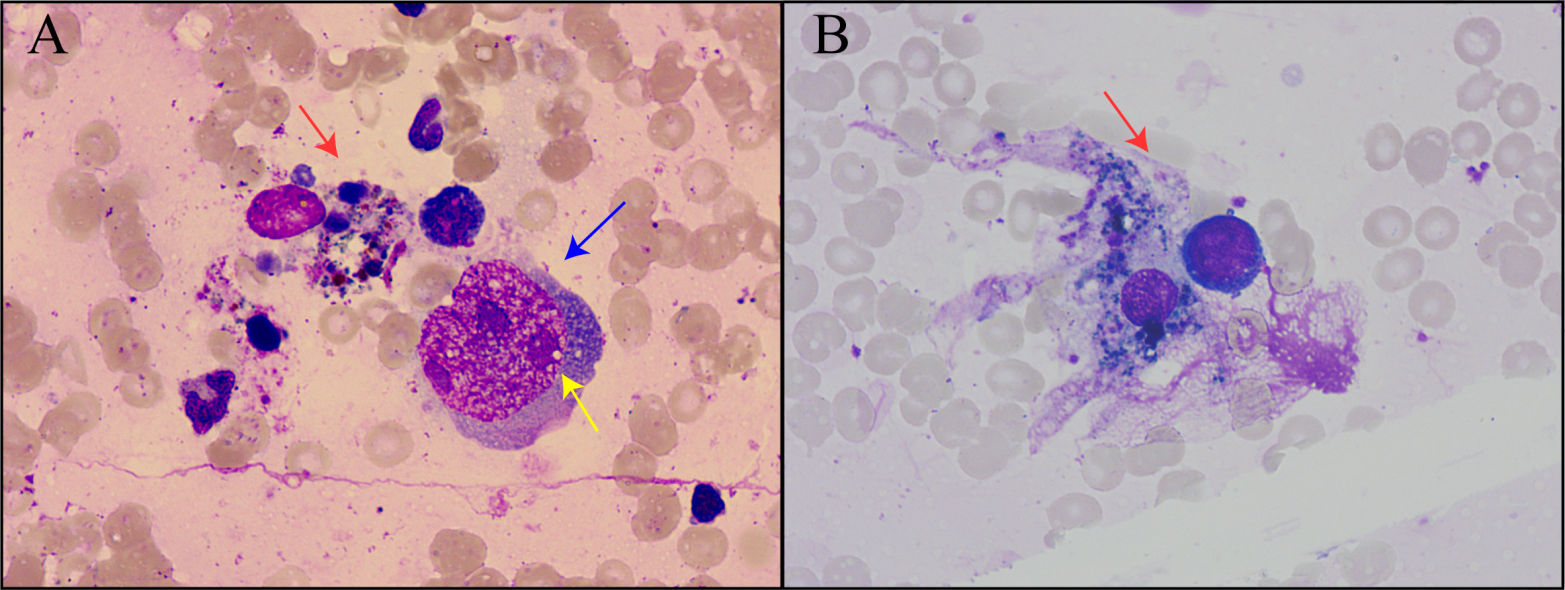
Bone marrow smear of the patient. **(A)** Hemophagocytic cells and proerythrocytes infected by parvovirus B19. Red arrows indicated hemophagocytic cells, blue arrows indicated proerythroblasts, and yellow arrows indicated viral inclusion bodies. **(B)** Hemophagocytic cells underwent phagocytosis.

**Figure 3:**
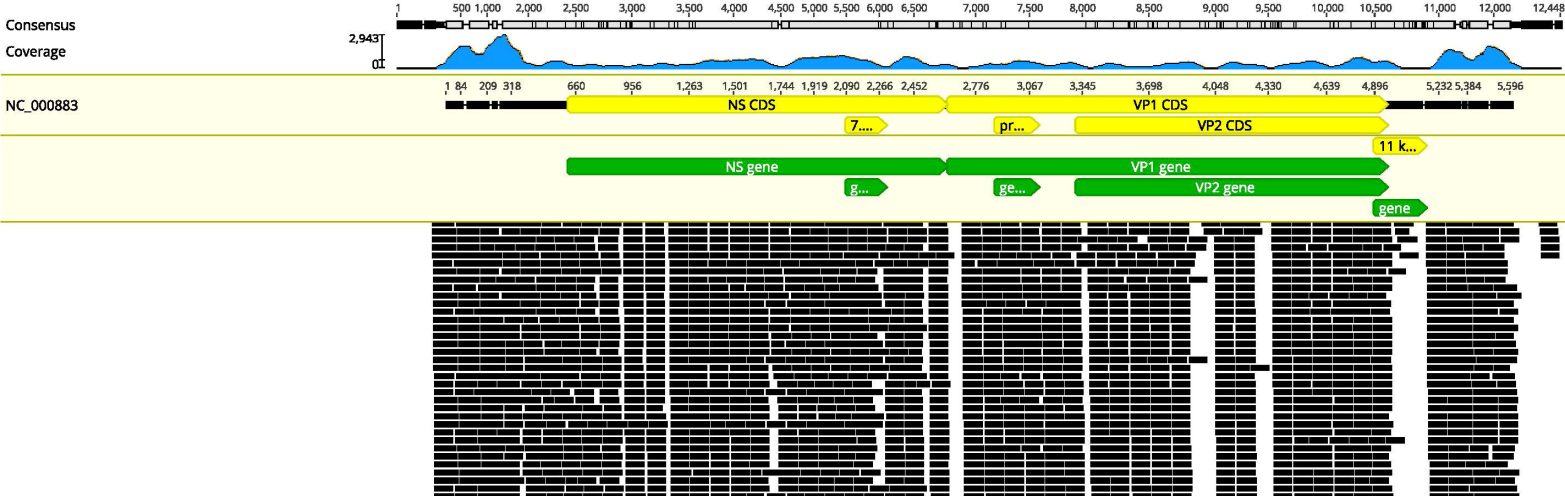
Results of next-generation sequencing in the peripheral blood. Mapping results of nucleotide sequences distributed along the genome of parvovirus B19 in the peripheral blood to parvovirus B19 reference genome NC_000883.

In this condition, bone marrow puncture was performed. The bone marrow smear revealed many giant proerythroblasts (**Figure 2A**) and erythroid maturation arrest. Basophilic, vacuolar cytoplasm and purple-colored virus inclusion bodies in the nucleus were observed in giant proerythroblasts suggestive of B19 infection. Next-generation sequencing of her peripheral blood confirmed that the only pathogen was parvovirus B19 (**Figure 3**), and quantitative PCR revealed that the viral load was 1.4×10^10^ copy/mL (range, 0-10^3^ copy/mL). Patient found to have increased ferritin (3865 ng/mL, reference range 7-323 ng/mL), triglycerides (4.6 mmol/L, reference range 0.3-1.7 mmol/L), reduced fibrinogen (0.83 g/L, reference range 2.0-4.0 g/L), elevated body temperature (38.5°C) for ten days, hemophagocytic cells in the bone marrow smears (**Figures 2A, B**), enlarged spleen, and cytopenia. Except for the unexecuted assay of serum soluble IL-2R and NK cell activity, the patient’s clinical manifestations met the diagnostic criteria of HLH as described (23).

The patient’s peripheral blood did not reveal pathogens other than parvovirus B19 detected by metagenomic next-generation sequencing as described before (27, 28). Also, whole exome sequencing did not identify any HLH-associated mutations. Other possible causes for HLH, including immune disorder and tumor were ruled out, and the patient was eventually diagnosed with parvovirus B19 infection complicated by HLH. After intravenous immunoglobulin (20g/d) for ten days, the patient’s serum IgG increased from 670mg/dL (reference range 860-1740 mg/dL) at the beginning to normal, reticulocytes increased to 3%, and the viral load of parvovirus B19 was reduced to 9.2×10^4^ copies/mL. Another bone marrow smear demonstrated that erythroid maturation was recovered, and the features of HLH were in remission.

## Discussion

Parvovirus B19 infection is common, and the prevalence of IgG antibodies in the population increases with age (29). In most cases, the infection can be asymptomatic and self-limited. Erythema infectiosum or arthropathy occurs in healthy children or adults (29). In immunocompromised patients, bone marrow transplant recipients (10) or patients with hemopathy, the infection can lead to autoimmune hemolytic anemia, neutropenia, thrombocytopenia, acute pure red cell aplasia (PRCA), transient aplastic crisis (AC), and rarely HLH (30).

Published cases of parvovirus B19 complicated by HLH are summarized in **Table 1**. Hemolytic diseases such as hereditary spherocytosis (42–45), sickle cell disease (40), alpha thalassemia (47), glucose-6-phosphate dehydrogenase deficiency (46), and autoimmune hemolytic anemia (41) were the most frequently reported primary disease. Also, a third of patients were immunocompromised, including patients with acquired immune deficiency syndrome (50), autoimmune diseases (37–39), undergoing chemotherapy (48), and post-transplantation patients (24–26), which can lead to persistent parvovirus B19 infection and may cause pure red cell aplasia. Besides, parvovirus B19 infection complicated with HLH has been reported in otherwise healthy patients (34–36) or patients with pregnancy (31), alcoholic hepatitis (32), myocarditis (33), or Melkersson-Rosenthal syndrome (49). Of note, parvovirus B19-associated reactivation may occur in post-transplantation patients, and some patients will develop pure red cell aplasia and HLH (51). Thus, parvovirus B19 reactivation should be considered in transplant patients with decreased hemoglobin and reticulocytes without a clear cause. Giant proerythroblasts and purple inclusions in the nucleus on bone marrow smears are typical changes in pure red cell aplasia caused by parvovirus B19. If HLH occurs in such patients, it is necessary to rule out the possibility of other pathogens, such as Cytomegalovirus and Epstein-Barr virus (23).

**Table 1:** Reported cases of Parvovirus B19 infection complicated by HLH.

reference	*primary disease*	treatment for Parvovirus B19	treatment for HLH	responses	survival
(24)	kidney transplantation	IVIG 0.4g/kg for five days	not mentioned	remission	alive
(25)	kidney transplantation	IVIG (100g cumulative)	dexamethasone	remission	alive
(26)	kidney and pancreas transplant	IVIG 0.4g/kg for five days	not mentioned	remission	alive
(31)	Pregnancy	not mentioned	prednisolone	remission	alive
(32)	alcoholic hepatitis	IVIG	methylprednisolone	lack of remission	dead
(33)	Myocarditis	/	/	/	dead
(34)	Healthy	IVIG	VP-16, prednisolone	remission	alive
(35)	Healthy	no treatment	no treatment	remission	alive
(36)	Healthy	not mentioned	prednisolone	lack of remission	dead
(37)	systemic lupus erythematosus	not mentioned	methylprednisolone and cyclosporine	remission	alive
(38)	Purpuric rash	IVIG 1g/kg	not mentioned	remission	alive
(39)	polyarteritis nodosa	no treatment	no treatment	remission	alive
(40)	sickle cell disease	not mentioned	methylprednisolone	remission	alive
(41)	autoimmune hemolytic anemia	not mentioned	methylprednisolone	remission	alive
(42)	hereditary spherocytosis	no treatment	no treatment	remission	alive
(43)	hereditary spherocytosis	IVIG	not mentioned	remission	alive
(44)	hereditary spherocytosis	no treatment	no treatment	remission	alive
(45)	hereditary spherocytosis	not mentioned	HLH-2004 protocol	remission	alive
(46)	Glucose-6-phosphate dehydrogenase deficiency	no treatment	no treatment	remission	alive
(47)	alpha thalassemia (HbH disease)	not mentioned	dexamethasone	remission	alive
(48)	Secondary AML	IVIG	dexamethasone	remission	alive
(48)	Anaplastic large T-cell lymphoma	IVIG	dexamethasone, VP-16	remission	alive
(49)	Melkersson-Rosenthal syndrome	not mentioned	PE; HSCT; HLH-2004 protocol	lack of remission	dead
(50)	human immunodeficiency virus	IVIG for 5 days	HLH-2004 protocol	remission	alive

IVIG, intravenous immune globulin; HLH, hemophagocytic lymphohistiocytosis; PE, plasma exchange; HSCT, hematopoietic stem cell transplantation

Treatment for parvovirus B19 infection is primarily symptomatic with IVIG used in chronic infection with anemia. A five-day continuous IVIG at 400 mg/kg/day is suggested for patients with solid organ transplantation or other immunosuppression (52), and in this case, parvovirus B19 infection and HLH features were remissions after the treatment of IVIG at 20 g/day. Most patients with parvovirus B19 infection complicated by HLH can achieve remission *via* IVIG and/or steroids. In addition, 20 out of 24 patients survived, indicating a better prognosis of parvovirus B19-associated HLH compared to other types of HLH (**Table 1**).

The condition and treatment of transplant patients are complex, and the clinical manifestations of the disease can be very confusing. When these patients present with chronic anemia and cytopenia, clinicians need to be alert to parvovirus B19 infection complicated by HLH, which requires hematologists, infectious disease specialists, critical care medicine specialists, and immunologists to work together to develop a clinical diagnosis and treatment plan to avoid misdiagnosis and inappropriate treatment. IVIG can alleviate or cure parvovirus B19 infection complicated by HLH, and at the same time, patients can be protected from the side effects of HLH-2004 treatment (23). Parvovirus B19 infection easily recurs in transplant patients due to long-term immunosuppression (53), but patients in this condition can avoid death caused by HLH.

## Conclusion

In transplant patients receiving long-term immunosuppressive therapy, clinicians need to be aware of parvovirus B19 infection and associated risk for HLH. IVIG treatment can alleviate features of parvovirus B19-associated HLH without the need for more toxic or immunosuppressive therapies.

## Data availability statement

The raw data supporting the conclusions of this article will be made available by the authors, without undue reservation.

## Ethics statement

The studies involving human participants were reviewed and approved by The ethics committees of the First Affiliated Hospital of Zhejiang University School of Medicine, approved the study protocol. Written informed consent for participation was not required for this study in accordance with the national legislation and the institutional requirements. Written informed consent was obtained for the publication of this case report.

## Author contributions

All authors drafted the manuscript, prepared the figures and critically reviewed the final manuscript. All authors contributed to the article and approved the submitted version.
